# Species traits affect phenological responses to climate change in a butterfly community

**DOI:** 10.1038/s41598-021-82723-1

**Published:** 2021-02-08

**Authors:** Konstantina Zografou, Mark T. Swartz, George C. Adamidis, Virginia P. Tilden, Erika N. McKinney, Brent J. Sewall

**Affiliations:** 1 Department of Biology, Temple University, 1900 North 12th Street, Philadelphia, PA 19122 USA; 2 The Pennsylvania Department of Military and Veterans Affairs, Fort Indiantown Gap National Guard Training Center, Annville, PA 17003 USA

**Keywords:** Ecology, Environmental sciences

## Abstract

Diverse taxa have undergone phenological shifts in response to anthropogenic climate change. While such shifts generally follow predicted patterns, they are not uniform, and interspecific variation may have important ecological consequences. We evaluated relationships among species’ phenological shifts (mean flight date, duration of flight period), ecological traits (larval trophic specialization, larval diet composition, voltinism), and population trends in a butterfly community in Pennsylvania, USA, where the summer growing season has become warmer, wetter, and longer. Data were collected over 7–19 years from 18 species or species groups, including the extremely rare eastern regal fritillary *Speyeria idalia idalia*. Both the direction and magnitude of phenological change over time was linked to species traits. Polyphagous species advanced and prolonged the duration of their flight period while oligophagous species delayed and shortened theirs. Herb feeders advanced their flight periods while woody feeders delayed theirs. Multivoltine species consistently prolonged flight periods in response to warmer temperatures, while univoltine species were less consistent. Butterflies that shifted to longer flight durations, and those that had polyphagous diets and multivoltine reproductive strategies tended to decline in population. Our results suggest species’ traits shape butterfly phenological responses to climate change, and are linked to important community impacts.

## Introduction

Phenological changes are among the most noticeable responses by plants and animals to anthropogenic climate change^1–3^. Although some taxa may fail to respond, or respond in ways that are maladaptive^4^, others may undergo evolutionary change or respond via phenotypic plasticity^5^. Among animals, more pronounced changes and faster responses often arise in ectotherms than in endotherms^6^, likely because the increased ambient temperatures and changes in precipitation associated with climate change have more direct effects on the metabolic rates, activity patterns, and developmental rates of ectotherms. Among butterflies, which have become prominent ectotherm models, several general patterns are now clear: many species have advanced their date of first seasonal appearance^7–9^, prolonged their duration of seasonal activity^10^ and increased their number of generations per season^8^. Nonetheless, phenological changes in response to climate change are far from uniform^6^, and can vary in magnitude and direction even among species experiencing similar environmental conditions^11^.

Understanding variation in phenological response is an important scientific and conservation challenge, because phenological changes may determine which species’ populations are harmed—such as via trophic mismatches (e.g., via desynchronization with a host plant^12^), lack of sufficient time to breed one last generation (the “lost generation” hypothesis;^13,14^), or limitations on species’ capacities to mitigate extreme temperatures or drought (such as via aestivation/diapause^4^)—and which are able to respond rapidly and effectively to environmental change^15,16^ through modification of phenology to correspond to changing climatic conditions^17,18^. Further, a lack of understanding of inter-specific variation hinders scientists’ and managers’ ability to predict shifts in species’ phenology and complicates the identification and management of species at high risk from climate change.

Several studies have suggested that species-specific ecological traits may predict the extent and direction of phenological shift^2,19^. Specifically, the intensity of phenological responses to climate change may be influenced by species-specific ecological traits such as larval diets (e.g., more constrained activity window in woody plant feeders^8^), number of generations (e.g., increased numbers of generations in multivoltine species^8^ or the facilitation of another generation in univoltine species^20^), habitat use (e.g., earlier spring emergence in more open habitats^21,22^), adult thermoregulation behavior (e.g., high-temperature dwellers are expanding at the expense of low-temperature dwellers^6,15^), or seasonal occurrence (e.g., spring broods show more pronounced advances in flight times than late-season fliers^23^, but see^24^ ). Thus, analysis of phenological responses to climate change in the context of species‐specific ecological traits provides a potentially powerful framework for identifying vulnerable species^25–28^ and making more robust generalizations about species’ phenological responses to climate change^29^.

In this study, we sought to improve understanding of how butterfly phenology (timing and duration of the flight period) has shifted over time, and to evaluate how species’ traits (larval trophic specialization, larval diet composition, voltinism) mediate these phenological shifts. We also examined changes in local climatic variables (temperature, aridity, and growing degree days) during the study period and over recent decades, and we sought to evaluate the sensitivity of phenological variation to seasonal fluctuations in temperature. We further related species’ traits and phenological patterns with the butterflies’ long-term population trends. We focused on late-flying butterflies in a temperate grassland community in Pennsylvania, USA. Phenology was derived from standardized repeated surveys of butterflies during late spring, summer, and early autumn over 19 years (1998–2016) for the extremely rare eastern regal fritillary *Speyeria idalia idalia*, and over 7–10 years (most recent years ending in 2016) for 17 other studied organisms.

We expected butterflies overall to be sensitive to temperature variation and, in accordance with predictions for climate change, to gradually shift seasonal flight periods to begin earlier and last for longer durations over the years since the beginning of the study period^30^. We also expected, however, that phenological changes would be associated with species-specific diets and reproduction strategies, as follows. First, we expected less pronounced phenological changes (both seasonal timing and duration) over the years in butterfly species with more restricted larval diets (oligophagous feeders) than those with more generalized larval diets (polyphagous feeders). This is because the direct response of oligophagous species to abiotic conditions altered by climate change may be tempered by their high dependence on their host plants^1^. Second, we expected larval woody feeders to show more pronounced changes in the seasonal timing and duration of the adult flight period compared to larval herb feeders since herbs can produce fresh shoots throughout the season while leaves of woody plants are available only for a short time period^8,31^ and the newly flushing leaves of woody plants are increasingly appearing earlier in response to climate change^32^. Thus, woody feeders may track the earlier availability of fresh leaves of woody plants, and transition to the adult flighted stage earlier. We further expected that, because flowering plants used by adults may undergo more limited temporal shifts than the host plants used by larvae, these same woody-feeding butterflies would also increase the overall duration of their flight period. Third, we expected univoltine butterfly species (those with only one generation per season) to show less pronounced phenological shifts than multivoltine species, which generally exhibit more plastic responses to variable environmental conditions^33,34^, and which have the ability to respond to longer growing seasons with an increased number of generations in a season^35^. Fourth, we assumed that each organism would be under continuous selective pressure to match its phenology to environmental conditions, but that more specialized or less variable ecological traits would constrain genetic or plastic shifts in behavior. Thus, we expected that species exhibiting oligophagy and univoltinism would exhibit lower sensitivity to annual temperature fluctuations than other species^36^. Finally, butterflies may face divergent pressures while responding both to altered abiotic conditions and host plants that separately shift phenology with climate change^37^. Such separate pressures could trigger trophic mismatches in phenology^1,16^, which could reduce survival^38^ most acutely among specialized butterflies. Similarly, limitations on individual longevity, increased mortality, or an inability to add additional generations in a season could constrain univoltine species’ response to longer growing seasons. Thus, in the context of ongoing climate change, we expected populations of oligophagous and univoltine species would fare worse over time than polyphagous or multivoltine species.

## Results

### Changes in local climate

**Figure 1:**
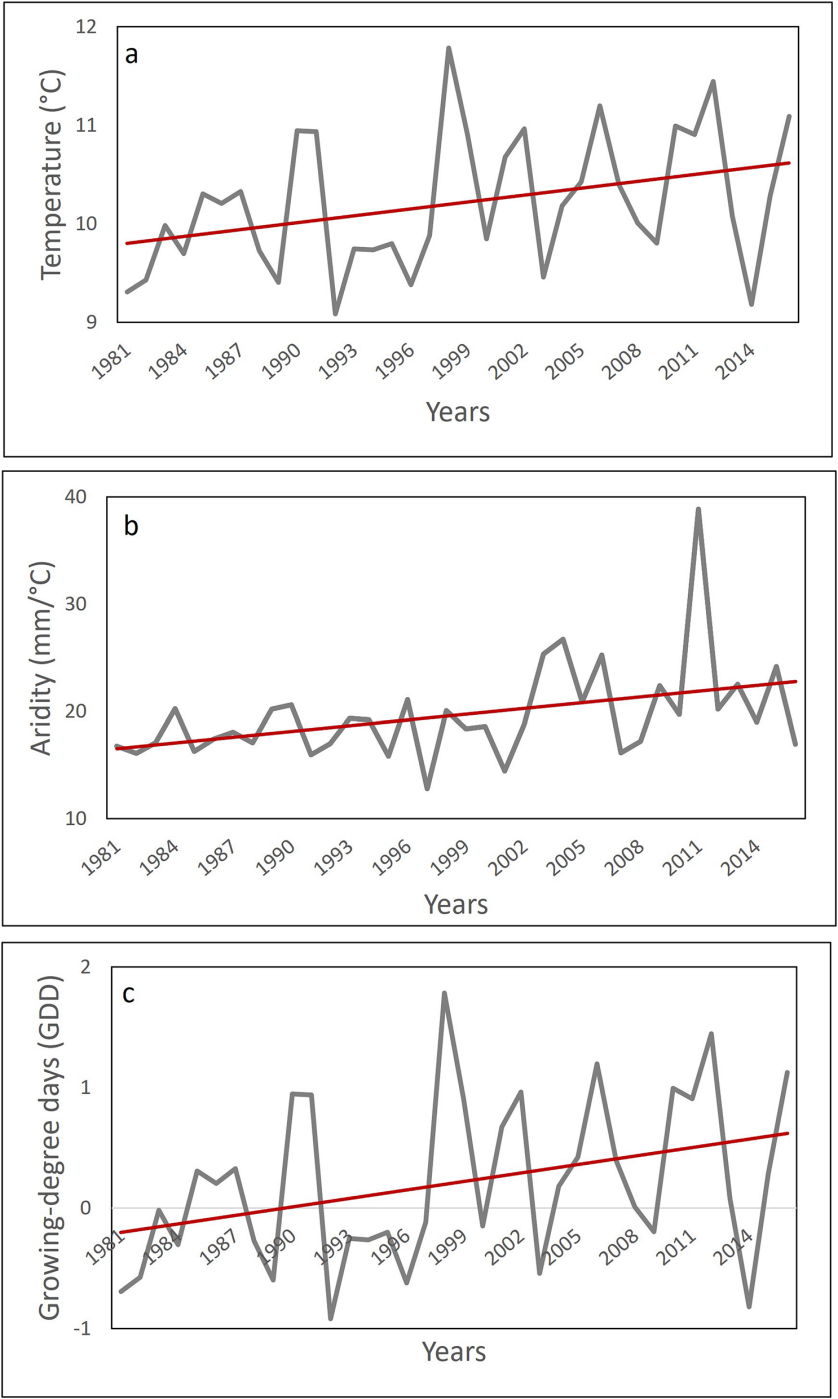
Positive trends in **(a)** mean annual temperature, **(b)** aridity, and **(c)** growing degree days across a 35-year period at Fort Indiantown Gap National Guard Training Center (FIG-NGTC). Note that for the Aridity index, the smaller the value the higher the level of aridity. Solid lines = annual change, dotted lines = linear regression line over time.

Over recent decades (1981–2016), the growing season at the field site became progressively warmer, wetter, and longer (Fig. 1). Mean annual temperature revealed a significant upward trend with time (Mann–Kendall Test; t = 149, *P* = *0.02*, Slope estimate = 0.02), with warming of about 0.7 °C over the 35-year period. The mean of the annual aridity index showed a significant upward trend (Mann–Kendall Test; t = 203, *P* = *0.002*, Slope estimate = 0.33), signifying progressively wetter conditions with an increase of 1.05 mm/ °C over the period. There was also a significant positive trend in growing degree days (GDD) (Mann–Kendall Test; t = 215, *P* = *0.001*, Slope estimate = 0.025), signifying a progressively longer growing season with an increase of 5.25 °C GDD over the period.

### Temporal trends in butterfly phenology and abundance

Applying individual median-based linear models we found that the majority (72%) of our organisms shifted either the mean date or the duration of their flight period or both (Table 1). Specifically, six of the 18 taxa (*Boloria bellona, Euptoieta claudia, Papilio glaucus, Papilio polyxenes,* both sexes of *S. idalia idalia,* and *Colias eurytheme/philodice*) advanced their mean flight date over time, while three others (*Phyciodes tharos, Polygonia interrogationis/comma, Satyrodes eurydice/Enodia anthedon*) showed some delay. Shifts in mean date were marginal in two of these taxa, *B. bellona* (*P* = *0.07*) and *P. interrogationis/comma* (*P* = *0.06*) (Table 1, Fig. S1). In addition, seven taxa prolonged the duration of the flight period (*Epargyreus clarus, Euptoieta claudia, Limenitis archippus, S. idalia idalia (F), P. interrogationis/comma, Satyrodes eurydice/Enodia anthedon, Speyeria cybele/aphrodite*) over time, while *Papilio troilus* shortened its duration (Table 1, Fig. S1). Shifts in duration were marginal in two of these taxa, *L. archippus* (*P* = *0.07*) and *P. interrogationis/comma* (*P* = *0.06*).

**Table 1:** Species traits of the 14 butterfly species and 4 species groups.

Species	LTS	LDC	Voltinism	Mean date	Flight period	Years	Mean date		Duration		Population trend	
							Slope	*P*	Slope	*P*	Slope	*P*
*Boloria bellona*	Oligo	Herbs	Multi	215	35	2007–2016	− 1.4	0.07	0.6	0.36	− 0.02	< 0.001
*Cercyonis pegala*	Poly	Herbs	Uni	207	21	2007–2016	0.1	0.76	− 0.3	0.1	0.02	< 0.001
*Colias eurytheme/philodice*	Poly	Herbs	Multi	213	24	2007–2016	− 1.39	0.01	− 0.3	0.38	0.06	< 0.001
*Epargyreus clarus*	Poly	Woody	Multi	210	24	2007–2016	− 0.18	0.22	0.7	0.01	0.1	< 0.001
*Euptoieta claudia*	Poly	Herbs	Multi	248	21	2007–2016	− 5.04	0.01	1.3	0.03	− 0.01	< 0.001
*Limenitis archippus*	Poly	Woody	Multi	224	27	2008–2016	0.005	0.94	1.2	0.07	0.09	< 0.001
*Limenitis arthemis astyanax*	Poly	Woody	Multi	206	28	2008–2016	3.07	0.14	1.08	0.4	0.1	< 0.001
*Lycaena phlaeas*	Poly	Herbs	Multi	220	12	2010–2016	− 0.06	0.83	0.08	0.95	(*)	
*Megisto cymela*	Poly	Herbs	Uni	163	15	2010–2016	− 0.48	0.22	− 0.5	0.37	0.2	< 0.001
*Papilio glaucus*	Poly	Woody	Multi	222	21	2007–2016	− 0.87	0.04	− 0.07	0.41	− 0.1	< 0.001
*Papilio polyxenes*	Poly	Herbs	Multi	240	10	2007–2016	− 2.76	0.02	− 0.02	0.96	− 0.07	< 0.001
*Papilio troilus*	Poly	Woody	Multi	217	18	2007–2016	0.21	0.95	− 0.4	0.001	− 0.1	< 0.001
*Phyciodes tharos*	Oligo	Herbs	Multi	221	20	2010–2016	3.36	0.01	0.4	0.22	0.3	< 0.001
*Pieris rapae*	Poly	Herbs	Multi	226	31	2007–2016	− 0.97	0.1	0.8	0.1	− 0.1	< 0.001
*Polygonia interrogationis/ comma*	Poly	Herbs	Multi	207	23	2010–2016	3.48	0.06	4.8	0.06	− 0.2	0.15
*Satyrodes eurydice/ Enodia anthedon*	Poly	Herbs	Uni	208	23	2008–2016	1.16	0.03	0.9	0.003	0.4	< 0.001
*Speyeria cybele/aphrodite*	Oligo	Herbs	Uni	210	24	2007–2016	− 0.11	0.5	0.4	0.02	0.06	< 0.001
*Speyeria idalia idalia (F)*	Oligo	Herbs	Uni	212	23	1998–2016	− 0.75	0.03	0.3	< 0.001	0.02	< 0.001
*Speyeria idalia idalia (M)*	Oligo	Herbs	Uni	190	14	1998–2016	− 0.59	< 0.001	− 0.08	0.74	0.04	< 0.001

Estimated shifts in mean flight date and duration of the flight period were obtained by fitting individual median-based linear models and population trends were developed on a model-based imputation approach.

*LTS* larval trophic specialization, *oligo* oligophagous (one food plant genus), *poly* polyphagous (> 1 food plant genus), *LDC* larval diet composition; herb feeders; woody feeders. Voltinism: number of generations per sampling period; uni: univoltine (one generation); multi: multivoltine (≥ 2 generations). Mean date: average of the weighted mean dates of appearance of each species as the latter has been calculated per year. Duration of the flight period: standard deviation of the mean date of species appearances. Population trend: change in the number of individuals observed per transect walk per year. *Speyeria idalia idalia*; *F* female, *M* male. (*) There is no available value due to model convergence.

**Figure 2:**
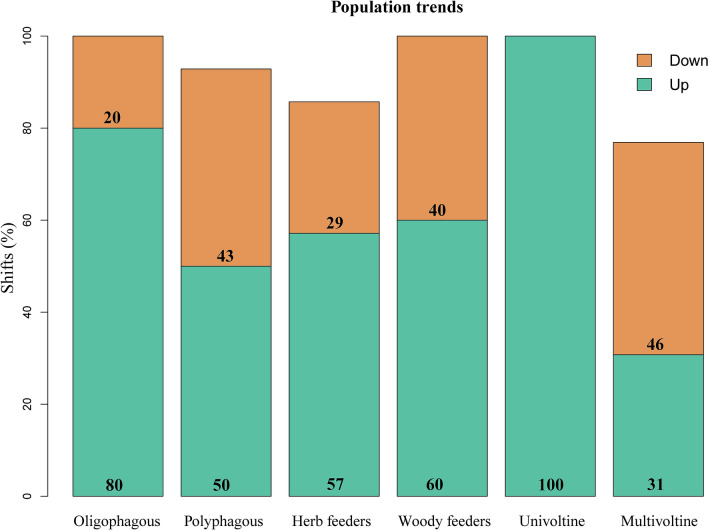
Population shifts grouped per ecological trait. Species for which model did not converge or population trend was not important are not shown. The two sexes of *S. idalia idalia* are treated as separate entities.

Population trends based on repeated counts at various sites were estimated on a model-based imputation model. Overall, populations were found to have an upward trend for most of the studied organisms (69%) (Table 1). However, when comparing assemblages by ecological traits, populations increased over time in a greater portion of oligophagous (80%) than polyphagous (50%) species, and a much greater portion of univoltine (100%) than multivoltine species (31%). (Table 1, Fig. 2). Further, all but one of the species found to have declined were both polyphagous and multivoltine (Table 1).

Surprisingly, phylogenetic regressions showed that there was a negative relationship between shifts in flight period and population trend (F-statistic: 5.8, slope = -0.06, *P* = *0.02*). That is, species that shortened flight duration over time tended to increase in population while species that lengthened their flight duration tended to decrease in population (Table 1). No relationship between shifts in mean date and population trend was detected (*P* > *0.05*).

### Sensitivity of species traits to phenological and temperature change

**Figure 3:**
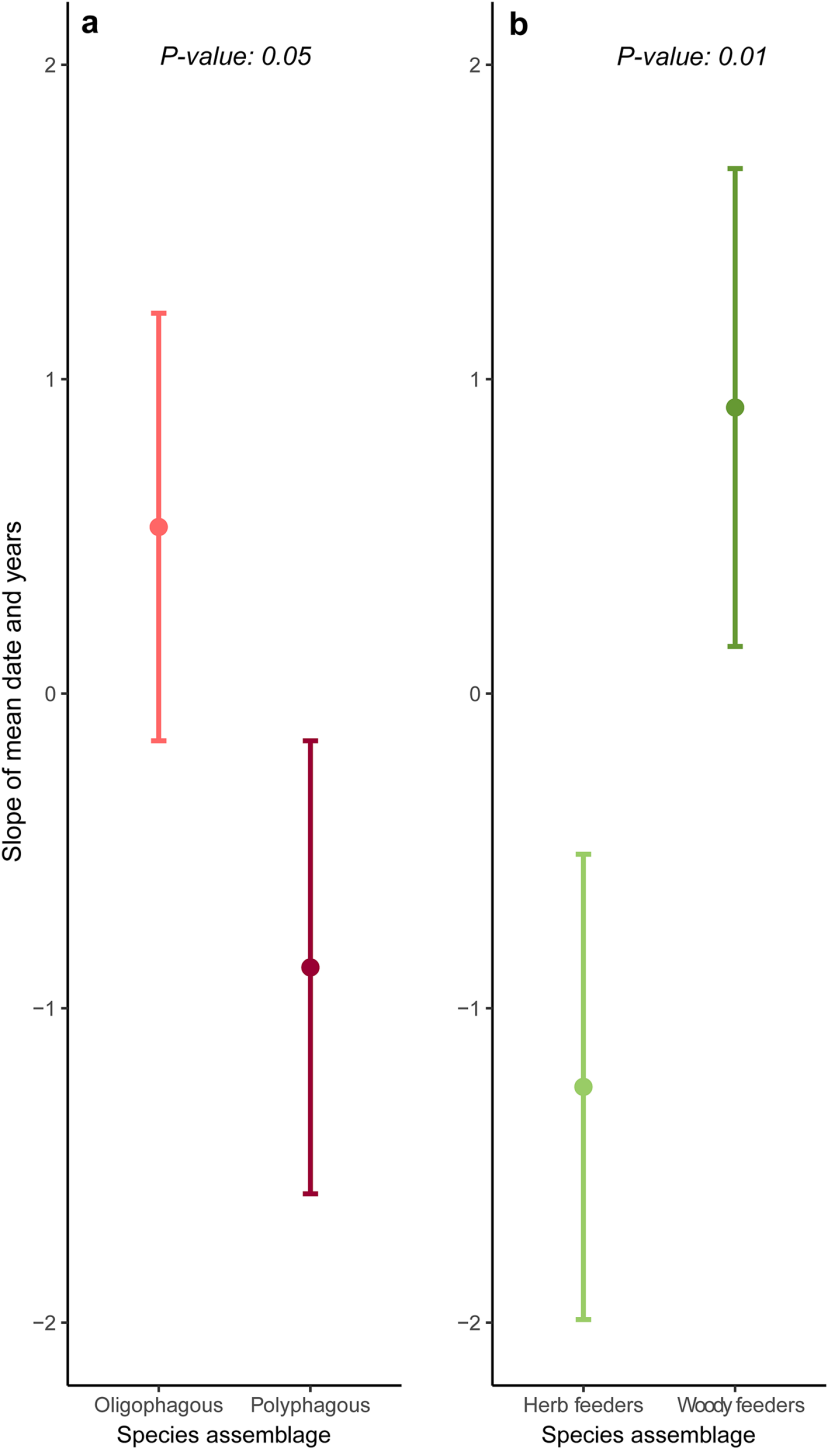
Sensitivity of larval trophic specialization (LTS) and larval diet composition (LDC) on shifts in mean date of appearance over the years. Error bars depict predicted mean values (circle) and standard error (upper and lower horizontal lines). LTS is shown on panel (**a**) (oligophagous in light red; polyphagous in deep red) and LDC is shown on panel (**b**) (herb feeders in light green; woody feeders in deep green).

**Figure 4:**
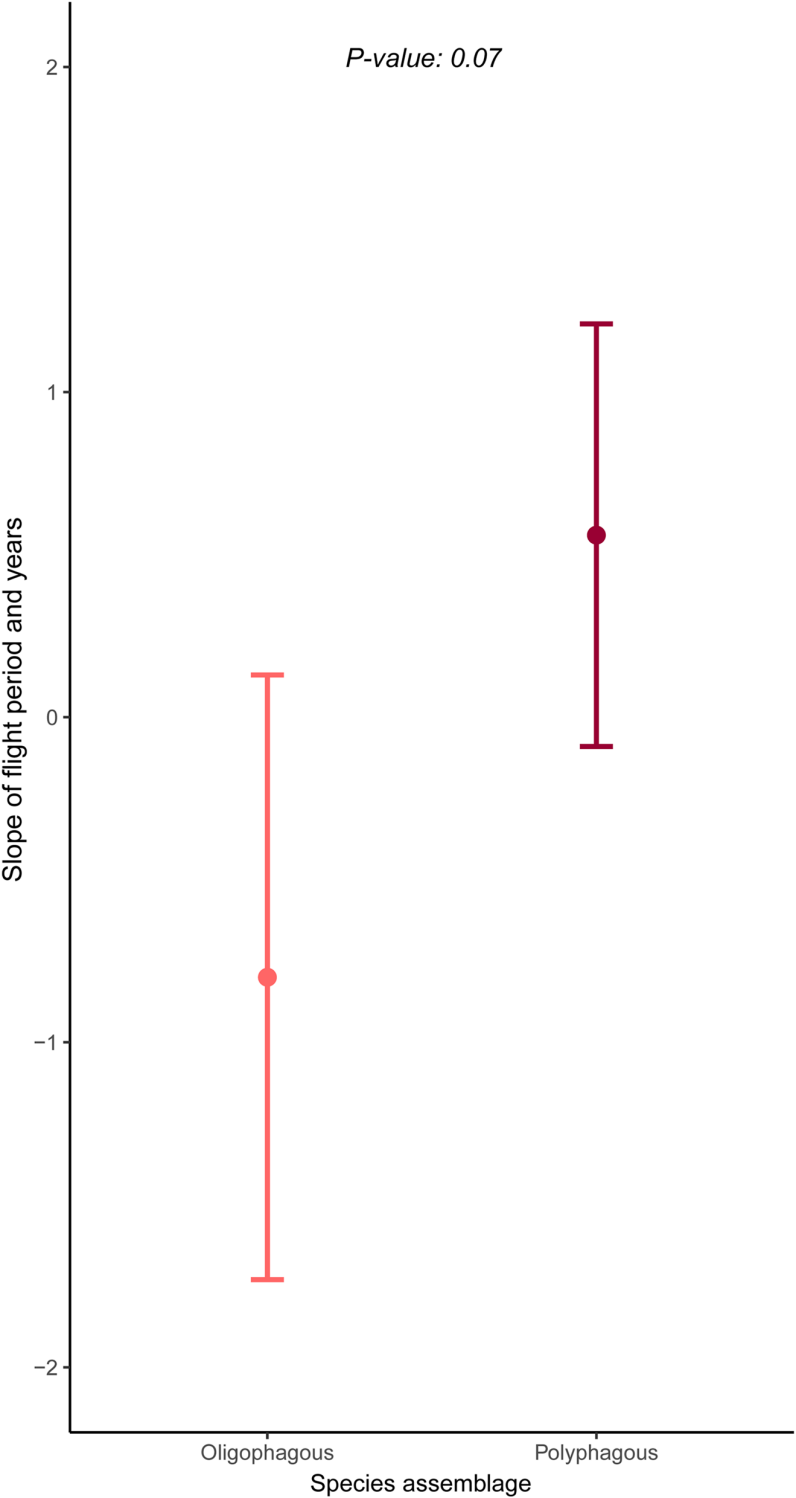
Sensitivity of larval trophic specialization (LTS) on shifts in duration of the flight period over the years. Error bars depict predicted mean values (circle) and standard error (upper and lower horizontal lines).

Phylogenetic regressions showed that species differing in larval trophic specialization increasingly differed in mean date over the years (F-statistic = 4.5, *P* = *0.05*), with oligophagous species increasingly flying later in the season and polyphagous species increasingly flying earlier (Fig. 3a, Table S1). Similarly, species differed by larval diet composition (F-statistic = 8.1, *P* = *0.01*), with woody feeders increasingly flying later and herb feeders increasingly flying earlier (Fig. 3b, Table S1). No relationship was detected between voltinism and mean date (*P* > *0.05*). Only a marginal difference in duration by larval trophic specialization was detected (F-statistic = 3.5, *P* = *0.07*), with a tendency toward shorter flight periods in oligophagous and longer flight periods in polyphagous species (Fig. 4, Table S1). No relationship was detected between larval diet composition and duration, or voltinism and duration (*P* > *0.05* in all cases).

**Figure 5:**
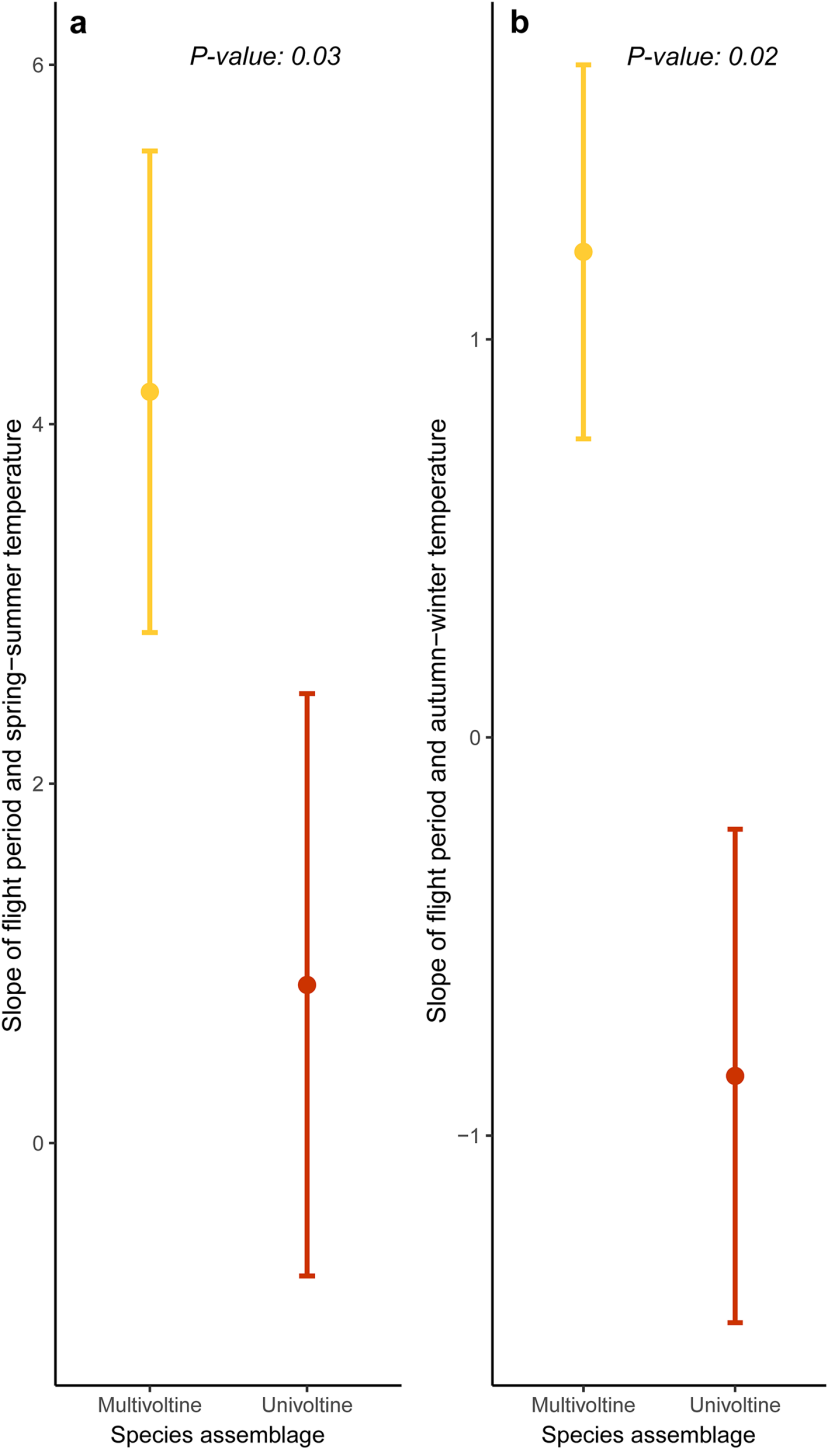
Sensitivity of voltinism on shifts in duration of the flight period over the seasonal fluctuations of temperature. Error bars depict predicted mean values (circle) and standard error (upper and lower horizontal lines). Slope of the relationship between flight period and spring–summer temperature is shown on panel **(a)** and slope of the relationship between flight period and autumn–winter temperature is shown on panel **(b)** (multivoltine in yellow; polyphagous in orange).

Lastly, species differed by voltinism in the extent to which the flight period was sensitive to the spring–summer (F-statistic = 5.8, *P* = *0.03*) (Fig. 5a, Table S1) and previous autumn–winter (F-statistic = 7, *P* = *0.02*) temperatures (Fig. 5b, Table S1). Specifically, multivoltine species substantially prolonged the duration of the flight period in response to warmer temperatures during both seasonal periods, and especially the spring–summer temperatures, while univoltine species slightly prolonged the flight period in response to warmer spring–summer temperatures but modestly decreased them in response to warmer temperatures in the previous autumn and winter (Fig. 5). No relationship was detected by larval trophic specialization or larval diet composition in the slope of duration versus either seasonal temperature (*P* > *0.05* in all cases). Likewise, no relationship was detected by any of the three ecological traits in the slope of mean date versus either seasonal temperature (*P* > *0.05* in all cases). Finally, no relationship was detected by any of the three ecological traits in either the slope of the mean date or the slope of duration versus annual (12-month) temperature (*P* > *0.05* in all cases).

## Discussion

The phenology of life history events represents a central mechanism by which species respond to environmental change, and recent shifts in phenology provide a crucial window into how species will respond to accelerating climate change. In this study, we sought to understand how butterfly phenology has shifted over time, how species’ ecological traits influence these shifts, and the consequences for butterfly populations. We found phenological shifts over time in 13 of the 18 butterfly taxa studied. Local increases in temperature, humidity, and the length of the growing season may suggest potential associations of the observed phenological shifts with climate change. We observed that shifts in mean flight date varied among assemblages differing in larval trophic specialization and larval diet composition. Shifts in flight duration also differed by larval trophic specialization. Further, species assemblages differed by voltinism in how flight duration was influenced by seasonal temperature. In addition, species with different ecological traits (larval trophic composition and voltinism) exhibited divergent population trends and shifts in flight duration were negatively associated with population trend. Together, these results suggest species’ ecological traits mediate the direction and intensity of climate-driven phenological shifts, and that these shifts have important consequences for population dynamics in the context of climate change.

### Phenological change by taxon

Phenological studies have mostly focused on early and spring flyers^9,39^, but here we demonstrate that flyers of late summer or summer broods of multivoltine species can also be responsive to climate change. First, 1/3 of the studied taxa advanced their mean flight date over time (by 0.59–5.04 days/year). Such phenological advances in the timing of butterfly flight are expected under warmer climate scenarios^9,10,40^. No changes were detected over time in half of the species. This may be because of limited time period (7–10 years) most species were observed. It may also be because we did not include early flyers in our analysis, which may have limited our ability to detect species responses during springtime, a period in which species responses are most pronounced^2,24,41^. A contrasting pattern, with a substantial delay in mean flight date over time, was observed in three taxa (1.16–3.48 days/year) and was most clearly evident in *P. tharos*. The distribution and flight behavior of this butterfly species is highly related to host plant availability (asters, milkweeds, thistles and sunflowers)^42^. However, no similar delays for species that use the same host plants (e.g. *P. glaucus, P. polyxenes*) were observed^37^. Another plausible explanation could be an extended aestival diapause because of the long photoperiod and high and increasing temperatures in the local summer climate.

Second, in accordance with expectations for warmer conditions^21^, seven of the butterflies prolonged their flight period (by 0.32–4.76 days per year). Of particular interest for conservation was that *S. i. idalia*, a globally threatened species that has begun to recover after a long population decline^43^, exhibited both a longer flight duration and earlier mean flight date. The lack of change we observed in the flight duration of ten taxa could again be attributed to the limited 7–10 year period that some of the studied organisms had been observed, but it is also consistent with the idea that local habitats can buffer ecological communities against coarse-scale trends and patterns of ongoing environmental change^22,44^. For instance, fine-scale habitat heterogeneity can create microrefugia, which may mediate species’ responses to climate changes^45^. In contrast to expectations, one species, *P. troilus*, shortened the duration of its flight period (0.4 days/year). Differences among taxa in observed phenological change may reflect not only direct effects of climate on butterflies, but also indirect effects of climate on the phenologies of host-plants^46^.

### The role of ecological traits in mediating phenological shifts over time

One key factor shaping changes in species’ phenology across years was larval nutrition. Oligophagous species delayed and shortened their flight periods while polyphagous species advanced and lengthened theirs. The expression of opposite phenological patterns suggests a different dynamic on species groups’ resilience to environmental change. For instance, species with generalized feeding habits have a wider niche and an ecological advantage to survive in areas with unpredictable and changing environment^47^. The large species pool used as food by polyphagous feeders may be a beneficial life history trait that allows organisms to maximize fitness as they track environmental change and respond via seasonally plastic strategies.

Another feeding trait influencing the magnitude of phenological change was larval diet composition. Herb feeders advanced their mean flight date whereas woody feeders showed moderate delays. Previous plant studies, including those ranging from the level of single individuals to entire biomes, have documented a clear advancement of leaf flushing in woody plants in the temperate zone in response to climate change^48^ but the response of leaf flushing is highly variable because woody plants also depend on cold temperatures to break bud dormancy^49^. If winter warming leads to insufficient bud dormancy^49^, woody plants will postpone the onset of newly flushing leaves^50^, also affecting the phenology of woody feeders that depend on their resources^51^. Moreover, at temperate latitudes, winter warming may increase intraspecific variation in leafing and flowering dates^52^. In addition, woody feeders often target the newly flushed leaves because of new leaves’ nutritional profile and/or less well-developed defenses^53^, but the association between plant palatability and shifts in phenology represents a largely overlooked pattern in the ecology of temperate butterflies. Together, these disparate plant responses to climate change could in turn alter butterfly community composition, by increasingly shifting the timing of herb and woody feeders’ life history events in the coming years.

### The influence of ecological traits in mediating phenological responses to seasonal temperature

Organisms often exhibit heterogeneity in their sensitivity to environmental factors^54^. It had previously been unknown, however, whether voltinism influenced the sensitivity of butterfly phenologies to seasonal temperature. We found multivoltine butterflies had high sensitivity to seasonal fluctuations in temperature, with consistent increases in flight duration in response to warmer seasonal temperatures, especially spring–summer temperatures. In contrast, univoltine species exhibited more modest shifts that increased flight duration with warmer spring–summer temperatures but decreased flight duration with warmer autumn–winter temperatures. These results accord with previous evidence for the higher responsiveness to climate of the multivoltine reproductive strategy. For instance, multivoltine species have greater synchrony in mean flight date across temperature gradients^45^, and they exhibit plastic phenological variation tied to annual climatic conditions and habitat use^22^. Further, warmer ambient conditions have increased the number of butterfly generations in dozens of temperate butterflies^55^, and can even facilitate a second generation in principally univoltine species^20^, where such species are constrained solely by the length of the growing season from having a second generation.

In our study, ecological traits were not otherwise associated with the relationship between phenology and seasonal temperatures. It should be noted, however, that phenology could be associated with variations in temperature that occur at a finer spatial or temporal scale than those measured in the aggregate with our site-level and season-long climate variables.

### Population consequences of climate and phenological changes

Earlier emergence or mean flight dates have been widely documented^1,9,15,31,56,57^ and have often been assumed to enable species to adjust to a changing climate. Shifts in mean flight dates were not related to population change across all species in our study, however. We did observe that, while not universal across all species, changes to flight duration were generally negatively related to changes in population over time. For instance, the *P. interrogationis/ comma* species group, which had the greatest increase in flight duration of any species (at 4.8 days per year), also had the largest population decline of all species (0.2 individuals observed per transect walk per year). The reasons for this pattern are unclear, but one potential mechanism could derive from the changing availability of floral resources at a community scale. In temperate zones, climate warming is expected to advance the phenology of early-flowering plants and delay it in late-flowering ones, and this divergent pattern of plant phenological shift could create a mid-summer gap in floral resources that might disproportionately affect species with longer summer activity periods^37^.

Overall, we observed an increasing population trend for most (69%) of our species pool, though percentages differed by assemblage: high portions of oligophagous and univoltine species exhibited population increases, while declining species were almost always both polyphagous and multivoltine. This contrasted with previous expectations that the greater plasticity exhibited by generalist feeders and via a reproductive strategy with multiple broods per season would confer an ecological advantage in a variable environment, and that this advantage would extend to directional environmental change caused by climate change^47^. For multivoltine species, one possible explanation is the lost generation hypothesis: if an extended growing period leads juvenile stages to complete development in late summer instead of entering the over-wintering stage, the chance of losing this generation is high and population declines may occur^14,58^. The declines we observed among multivoltine species further highlight that species exhibiting greater responsiveness to climate do not necessarily fare better than less responsive ones, at least in the short term. In contrast, evolved strategies in univoltine species, such as the use of summer aestivation, may have allowed them to effectively respond to longer periods of thermal stress and drought, and to climate-driven shifts in plant phenology.

Even so, it is unclear whether strategies that have thus far benefitted univoltine butterflies will continue to prove beneficial as climate change accelerates. For instance, in univoltine species like *Maniola* or *Speyeria* butterflies, in which important life history events like summer dormancy or oviposition are induced by photoperiod^59^, shifts in plant phenology and climate might lead to an increasing temporal mismatch with optimal conditions for growth, survival, and reproduction^58^. Alternatively, the use of extended aestivation to adjust to a warming climate could incur several costs, including shortened activity periods needed for mating or to meet nutritional needs prior to entering aestivation, increased susceptibility to predation or disease during the longer aestivation period, or heightened survival risk if the increasingly warmer or drier conditions during aestivation exceed the butterflies’ environmental tolerances^4^.

## Methods

### Study site

Data were collected at Fort Indiantown Gap National Guard Training Center (FIG-NGTC), a military training area in southeastern Pennsylvania, USA (40°26′13.15′'N, 76°34′33.8′'W). The landscape consists of a mosaic of forests, semi-natural grasslands, and rangelands heavily used for military training. FIG-NGTC extends over 6920 ha, with altitudes from 110 to 437 m above mean sea level^60^. We focused on grassland habitat (88.22 ha) within this mosaic.

The climate is humid continental with February the driest and May the most humid month. The grassland habitat of FIG-NGTC is of particular interest for conservation both because it hosts a diverse native butterfly community and because it harbors the only remaining viable population of an extremely rare butterfly, *S. i. idalia*^61,62^, which is Critically Imperiled^63^.

### Butterfly species

For 19 years (1998–2016), counts of *S. i. idalia* were made weekly along five fixed routes in grassland habitats by trained biologists and volunteers, following the Pollard walk transect method^64^. Since *S. i. idalia* is a large, sexually-dichromatic butterfly that is detectable and can be identified to species and sex from a distance, surveys focused on an 18.3 m (20 yards) band on either side of the transect. Sampling included the months of June to September in all years, but sampling sometimes began earlier (in May) or extended later (until October), when weather conditions were suitable for butterfly activity. Beginning in 2007 and again in 2008, additional species and species groups were recorded during transect walks, with the same methods. From 2010 onwards, sampling was further extended to all identifiable species or species groups in the butterfly community. While this yields observations of just 7–10 years in most species, butterflies have been found to respond to climate change over similar time frames in other studies^15,65^.

These butterfly surveys resulted in a monitoring dataset that included 41 species or species groups (sets of species with similar appearance lumped together in field data because they could not be properly distinguished visually at a distance) that were consistently identifiable during surveys. From this dataset, we removed observations that could contribute to bias, as follows. First, we removed species groups if different species within the same group had different traits of interest (6 species groups excluded). The species groups that were retained were always pairs of species and these pairs were more closely related to each other than to other taxa in this analysis. Second, to avoid trying to draw conclusions from small sample sizes, we removed species that were observed infrequently (≤ 4 counts per year or ≤ 4 years of records) from analyses (16 species). Third, we excluded an early univoltine flyer for which the emergence day was before sampling began. For multivoltine species included in the analysis we also ignored the first flight period of each year (which sometimes was also before sampling), and focused on the second flight period, which always fell during the sampling period. For *Epargyreus clarus* the mean date and duration are calculated from the total time period encompassing the two flight periods. The resulting dataset included 14 species and 4 species groups (hereafter, “taxa”; Table 1). The dataset also included separate data on both sexes of *S. i. idalia* because, unlike all other species in this analysis, a different phenology characterizes each sex^43^. Males emerge in June and die after mating in July, while females emerge in July and after a summer dormancy, they oviposit their eggs from late August to mid-September and then die.

### Species traits

For each of these 18 taxa, we then determined three life-history traits that are likely correlated with changes in flight period in response to climate change^40,56^. These included (i) larval trophic specialization (oligophagous *versus* polyphagous)^8^ ; (ii) the larval diet composition (woody *versus* herb feeders)^40^ ; (iii) voltinism (univoltine *versus* multivoltine species)^25,40^ (Table 1). Data on these life history traits were primarily from field observations by the authors and secondarily from field guides^66,67^.

### Phenology variables

Two phenological variables were calculated for each butterfly species each year. The first variable, the weighted mean date of adult flight (“mean date” hereafter) was calculated as:

$${\sum }_{t=1}^{J}{{p}_{k}t}_{, } {p}_{k}=\frac{{n}_{k}}{{N}_{k}}$$

where *p*_*k*_ is the relative abundance of species (*n*_*k*_ is the number of individuals of species *k* per visit, *N*_*k*_ is the total number of individuals of species *k* per year), *t* is the date in Julian days (1 = January 1st), and *J* is the last observation date^56^. The second variable, the duration of the flight period, was calculated as the SD about the mean date^9,68,69^. These variables are less sensitive to sampling effort, extreme events, and population trends than variables like first observation or time between first and last observation^9,21,70^.

### Butterfly phylogeny

Traits of closely related taxa such as food source preferences, number of broods and temporal sensitivities to temperature fluctuations may be similar due to common ancestry and hence statistically dependent in comparative analyses^71^. In order to account for phylogenetic relatedness we used the ultrametric phylogenetic tree published by^72^ to construct a phylogenetic tree of our species pool (Fig. S2, Table S2).

### Climate variables

First, to investigate whether there has been a significant directional change in local climate during the last decades, a 35-year period (1981–2016) was considered. Meteorological data were obtained for the precise location of the study area from the PRISM climate model^73^, which estimates temperature and precipitation variables for the continental United States, as interpolated from multiple neighboring weather stations while controlling for physiographic variables^74^. Climate variables examined were annual temperature (Temperature), aridity index (Aridity) and growing degree-days (GDD). Aridity index is a measure of drought, calculated as P/2T, where P is mean annual precipitation (mm) and T is mean annual temperature (°C)^75^. Note that the higher the aridity index value, the less arid the conditions. Cumulative seasonal heat units, expressed as growing-degree days (GDD), were used as an indirect measure of the effect of temperature on butterfly populations. The GDD value represents the accumulated number of degrees over a season where the average daily temperature is greater than a threshold temperature [(Temperature_max_ + Temperature_min_)/2 − Temperature_threshold_]; this measure is known to contribute to organismal development^76^. Here the threshold was set as 10 °C, the minimal thermal condition conducive to butterfly development and survival^40,57^.

Then, to document the sensitivity of butterfly phenology to abiotic factors, we considered the primary sampling period in this study (2007–2016) and we estimated the three following climatic cues (Donoso et al. 2016): (1) a 12-month period starting with the month after the last butterfly survey (i.e., beginning in October), (2) the spring–summer (March–August) period that overlapped with the annual surveys, and (3) the autumn–winter (September—February) period prior to each annual survey.

### Statistical analysis

To detect possible trends in each one of the climate time series we ran trend analyses. The significance of the trend was assessed by a nonparametric rank-based test (Mann–Kendall test) and evidence for an increasing trend versus the null hypothesis (no trend) was tested at a 0.95 level of confidence.

Next, to evaluate changes in species’ phenology over the study period, we assessed each taxon separately with median-based linear models, as this method is robust to outliers^77^. Time was the explanatory variable, and phenological descriptors were the responses. Positive slopes indicated mean flight date was later or duration of the flight period was longer in more recent years for the species. We used mblm() function^78–80^ in R package NSM3^81^. Diagnostic graphs for residuals independency and homogeneity and the Shapiro–Wilk test for normality were used to check if models’ assumptions were met. In addition, we estimated population trends for each taxon. Annual counts were modeled as a function of the fixed effects of “time” (years) and “site” (a suite of five sampling grasslands) using the trim() function in the R package *rtrim*^82^. Sampling effort was similar across months, sites, and years^83^.

Furthermore, we evaluated whether butterflies responding appropriately to climate change (via a shift to an earlier date or a shift to longer duration) would benefit with increased population numbers (or at least remain stable) while butterflies that showed minimal or no shifts would suffer more from the effects of climate change. We therefore tested whether population trends and phenological shifts tended to co‐vary, while accounting for phylogenetic relatedness as described below.

### Attributing sensitivity of species traits to shifts in phenology and seasonal temperature

We then used a phylogenetic analysis to evaluate how butterfly phenology has changed over time and in response to seasonal temperature fluctuations, and to determine whether the direction and magnitude of those changes is associated to specific ecological traits of butterflies. First, we regressed the mean date and duration by year for each of the 18 taxa, and then we included the slopes of those regressions (one per taxon) as a response variable in a comparative analysis. Then, we grouped our species pool into subsets of species (hereafter, species assemblages) on the basis of their ecological traits: (1) larval trophic specialization (oligophagous versus polyphagous species), (2) larval diet composition (woody versus herb feeders) and (3) voltinism (univoltine versus multivoltine species).

We used phylogenetic generalized least squares (PGLS) to test for an association between mean date of appearance/duration and species traits using the gls() function in R package *nlme*^84^. The slope between the mean date or duration and years was treated as response variable and species traits as categorical variables. The PGLS approach allowed us to examine relationships using the most common models for evolutionary change (Pagel's lambda, Brownian motion, Ornstein–Uhlenbeck)^85^. We compared model fit using Akaike’s Information Criterion (AIC) and used the model with the best fit to estimate linear regression associations. Furthermore, to test the sensitivity of butterfly phenology to climatic cues, we first calculated the relationship (the slope between mean date or duration and seasonal temperature), and then we included the relationship in the comparative analysis described earlier. To account for phylogenetic structure, we also fit PGLS trait evolution models and compared model fit using Akaike’s Information Criterion.

## Supplementary Information

Supplementary Information.
