# Supplementary material for: Species traits affect phenological responses to climate change in a butterfly community
Source: Sci Rep. 2021 Feb 8;11:3283. doi: 10.1038/s41598-021-82723-1 (PMC7870830; doi:10.1038/s41598-021-82723-1)
Supplement: Supplementary file 1 — Supplementary Information. [file 41598_2021_82723_MOESM1_ESM.docx]

*Journal: Scientific Reports*

**Supplementary Information**

Title: Species traits affect phenological responses to climate change in a butterfly community

Konstantina Zografou, Mark T. Swartz, George C. Adamidis, Virginia P. Tilden, Erika N. McKinney, Brent J. Sewall

**Table S1**. Phylogenetically corrected association of species traits with phenology (mean date, flight period) along years and over seasonal temperature fluctuations using phylogenetic generalized least squares (PGLS).

| **Response variable** | **Predictors** | **Species assemblages** | **Predicted means** | ***P-value*** | **Phylogenetic model** |
| --- | --- | --- | --- | --- | --- |
| Slope of the relationship between mean date and years | LTS | Oligophagous | 0.53 | **0.05** | PL |
|  |  | Polyphagous | -0.87 |  |  |
|  | LDC | Herb feeders | -1.25 | **0.01** |  |
|  |  | Tree feeders | 0.91 |  |  |
| Slope of the relationship between the flight period and years | LTS | Oligophagous | -0.80 | 0.07 | PL |
|  |  | Polyphagous | 0.56 |  |  |
| Slope of the relationship between the flight period and spring-summer temperature | LTS | Oligophagous | 3.61 | ns | BM |
|  |  | Polyphagous | 1.44 |  |  |
|  | Voltinism | Univoltine | 0.88 | **0.03** |  |
|  |  | Multivoltine | 4.18 |  |  |
| Slope of the relationship between the flight period and autumn-winter temperature | LTS | Oligophagous | 0.50 | ns | OU |
|  |  | Polyphagous | -0.12 |  |  |
|  | Voltinism | Univoltine | -0.85 | **0.02** |  |
|  |  | Multivoltine | 1.22 |  |  |

Significant associations (P < 0.05) are in bold. Preferred model for phylogenetic residual error in PGLS analyses are best fit models chosen by model selection among Pagel's lambda (PL), Brownian motion (BM) and Ornstein–Uhlenbeck (OU) models. LTS: Larval Trophic Specialization; LDC: Larval Diet Composition


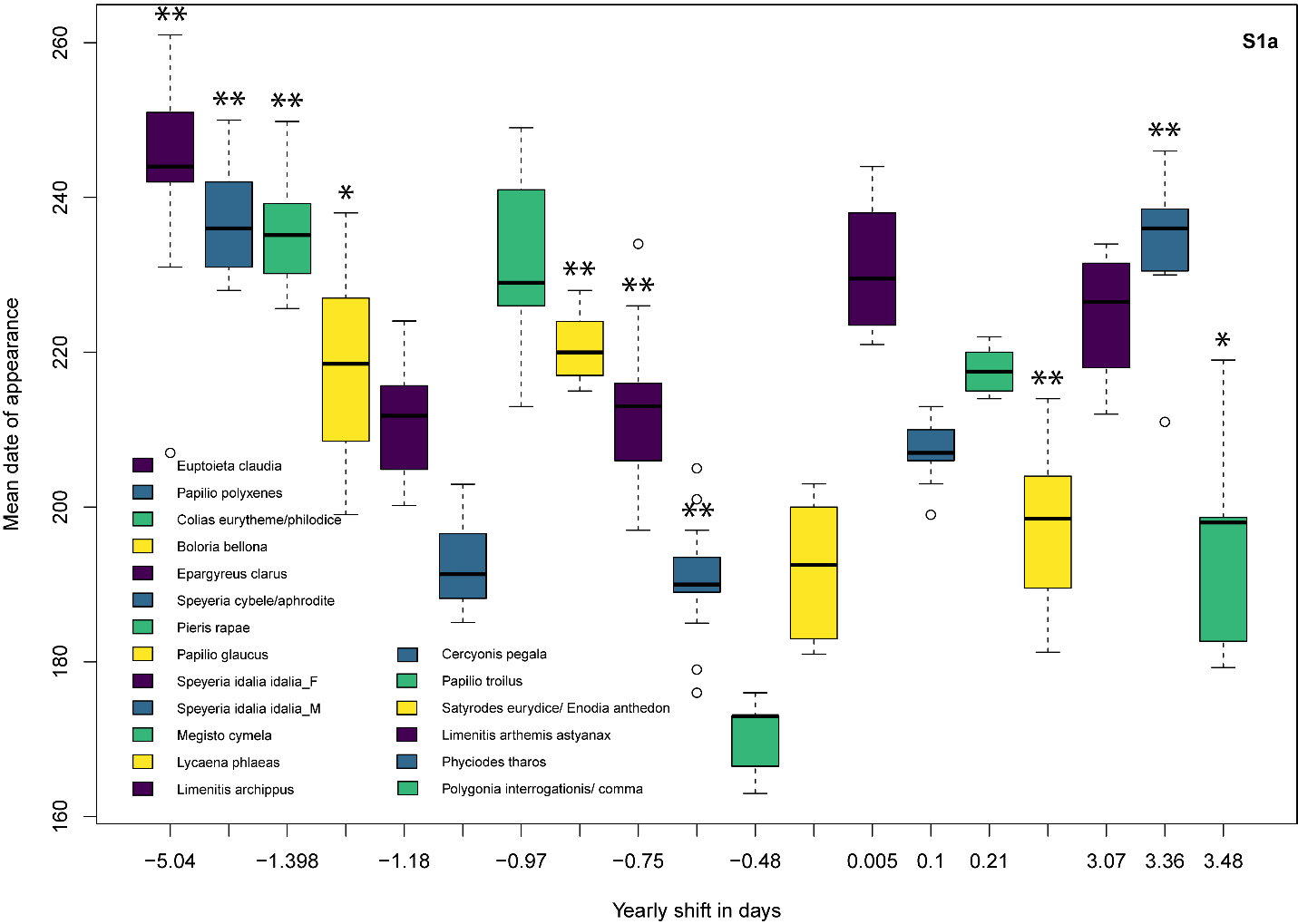


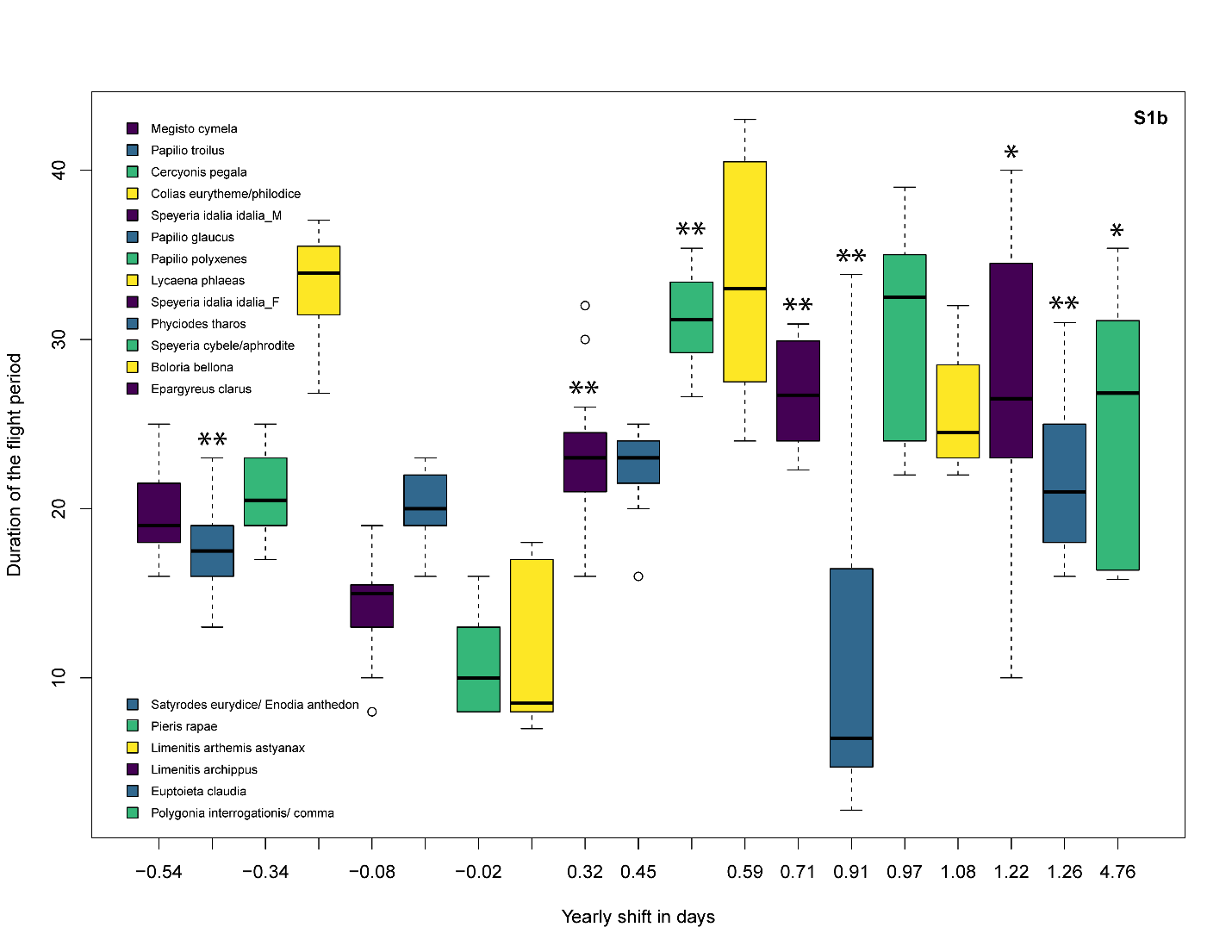


**Figure S1**. Boxplots representing the shift our studied organisms underwent over the years of this study (note that the number of years varied per species) in terms of (a) mean date of appearance and (b) duration of the flight period. An asterisk (*) corresponds to marginal changes and two asterisks (**) to significant changes. For *Speyeria idalia idalia*, F = female, M = male.


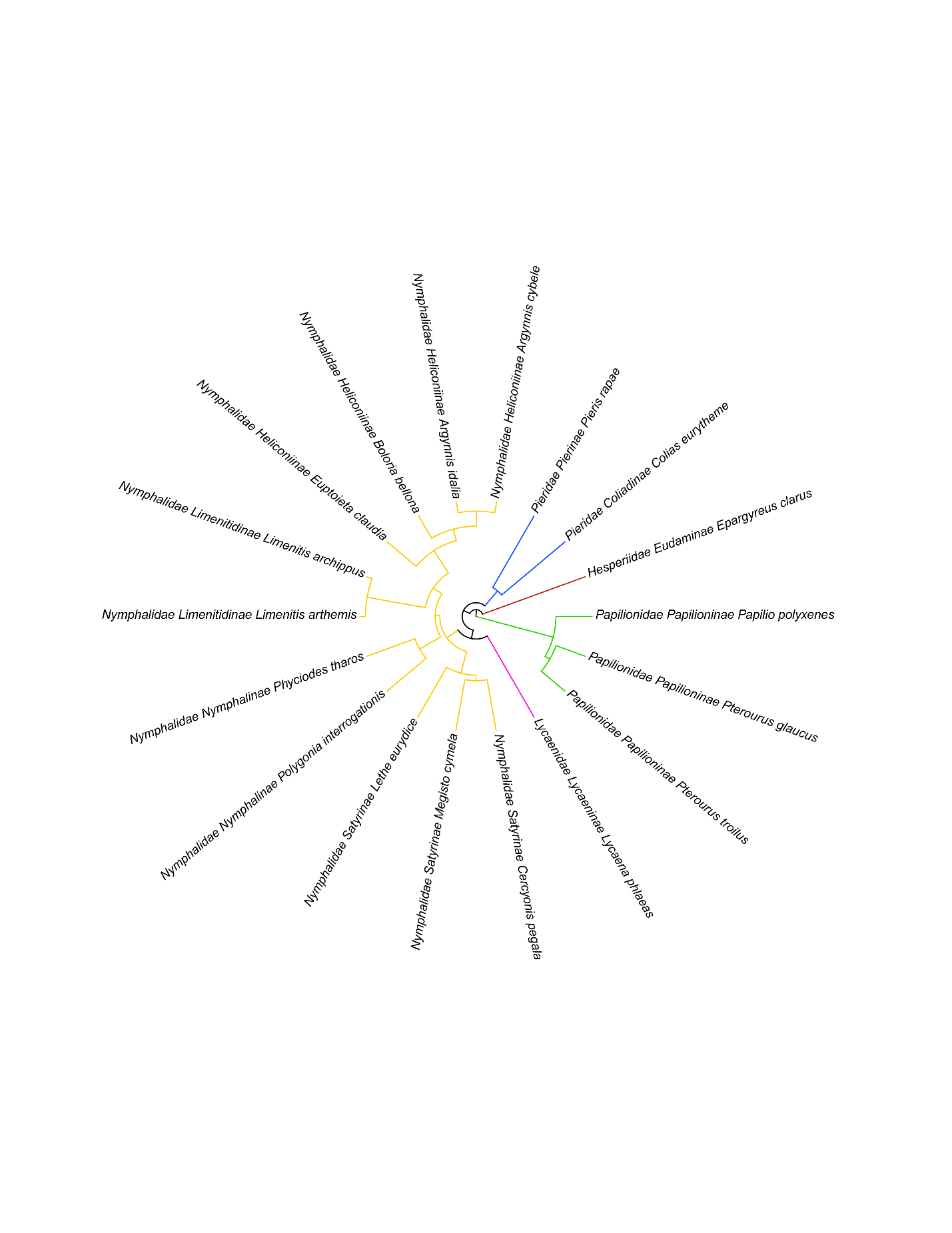


**Figure S2.** Maximum likelihood reconstruction based on COI sequences for the 18 butterfly species and species groups found in Fort Indiantown Gap National Guard Training Center, Annville, USA. The different families are represented by different colors (in taxonomic order and proceeding counterclockwise: Papilionidae, green; Hesperiidae, red; Pieridae, blue; Nymphalidae, orange; Lycaenidae, fuchsia).

For the construction of Figure S3 we utilized the phylogenetic tree that was built by Earl et al. 2020 ^1^ for the butterflies of North America. In the four cases of species groups (sets of similar species lumped together in field data because they could not be properly distinguished visually at a distance) we chose arbitrarily the first out of the two referred to in the species list (**Table S2**). Valid names of the phylogenetic tree by Earl et al. 2020^1^ were derived from a global checklist ^2^.

**Table S2.** The list of species used for producing Figure S3. For the four cases where species groups are presented, the underlined species (*Colias eurytheme, Speyeria cybele, Polygonia interrogationis, Satyrodes eurydice*) in those groups are the selected names presented in **Figure S2**.

| **No** | **Species nomencluture as presented in Earl et al. 2020** | **Our species list** |
| --- | --- | --- |
| 1 | Hesperiidae_Eudaminae_Epargyreus_clarus | *Epargyreus clarus* |
| 2 | Pieridae_Coliadinae_Colias_eurytheme | *Colias eurytheme/philodice* |
| 3 | Pieridae_Pierinae_Pieris_rapae | *Pieris rapae* |
| 4 | Nymphalidae_Heliconiinae_Argynnis_cybele | *Speyeria cybele/aphrodite* |
| 5 | Nymphalidae_Heliconiinae_Argynnis_idalia | *Speyeria idalia* |
| 6 | Nymphalidae_Heliconiinae_Boloria_bellona | *Boloria bellona* |
| 7 | Nymphalidae_Heliconiinae_Euptoieta_claudia | *Euptoieta claudia* |
| 8 | Nymphalidae_Limenitidinae_Limenitis_archippus | *Limenitis archippus* |
| 9 | Nymphalidae_Limenitidinae_Limenitis_arthemis | *Limenitis arthemis astyanax* |
| 10 | Nymphalidae_Nymphalinae_Phyciodes_tharos | *Phyciodes tharos* |
| 11 | Nymphalidae_Nymphalinae_Polygonia_interrogationis | *Polygonia interrogationis/ comma* |
| 12 | Nymphalidae_Satyrinae_Lethe_eurydice | *Satyrodes eurydice/ Enodia anthedon* |
| 13 | Nymphalidae_Satyrinae_Megisto_cymela | *Megisto cymela* |
| 14 | Nymphalidae_Satyrinae_Cercyonis_pegala | *Cercyonis pegala* |
| 15 | Lycaenidae_Lycaeninae_Lycaena_phlaeas | *Lycaena phlaeas* |
| 16 | Papilionidae_Papilioninae_Pterourus_troilus | *Papilio troilus* |
| 17 | Papilionidae_Papilioninae_Pterourus_glaucus | *Papilio glaucus* |
| 18 | Papilionidae_Papilioninae_Papilio_polyxenes | *Papilio polyxenes* |

**References**

1 Earl, C. *et al.* Spatial phylogenetics of butterflies in relation to environmental drivers and angiosperm diversity across North America. *bioRxiv*, 2020.2007.2022.216119, doi:10.1101/2020.07.22.216119 (2020).

2 Lamas, G. Checklist : Part 4A. Hesperioidea-Papilionoidea. *Atlas of neotropical lepidoptera*, 1-439 (2004).
